# Neck circumference as a complementary measure to identify risk of excess body mass in children younger than 2 years old

**DOI:** 10.1186/cc14683

**Published:** 2015-09-28

**Authors:** Daniela dos Santos, Aila Aparecida Farias, Caroline Kroll, Katherinne B Wanis Figueirêdo, Marco Fábio Mastroeni, Mayte Bertoli, Sandra Ana Czarnobay, Silmara S de BS Mastroeni

**Affiliations:** 1 Universidade da Região de Joinville--Univille, Joinville, SC, Brazil

## Objective

To evaluate the effectiveness of neck circumference (NC) as a measure for assessing risk of excess body mass in children aged 13-24 months.

## Methods

From a total of 435 children born in 2012 in a public maternity hospital of Joinville, Brazil, 279 participated in a new investigation 1 year later. Body mass, length and NC were collected in their homes between March 2013 and March 2014. The best cutoff value for identifying overweight/obese children using the body mass index (BMI) was determined by the receiver operating characteristic curve (ROC curve), according to gender and the age groups: 13-15 months, 16-19 months and 20-24 months.

## Results

NC was positive and significant. The NC cutoff points increased with increasing age group in boys (23.6, 23.9 and 24.0 cm) and girls (23.4, 23.5 and 23.6 cm), respectively, for the 13-15, 16-19 and 20-24 age groups. See Tables 1, 2, 3, 4.

**Table 1:** General characteristics of the children according to BMI category and sex, Joinville, SC, Brazil, 2013-2014.

Characteristic	BMI ≤P85	BMI >P85	*p *value
Boys	(*n *= 82)	(*n *= 60)	
Age (months)	16.1 (3.1)	17.4 (3.1)	0.004
Body mass (kg)	10,433.1 (1124.7)	12,839.7 (1792.8)	<0.001
Length (cm)	79.8 (4.1)	81.1 (4.2)	0.063
BMI (kg/m^2^)	16.3 (1.0)	19.4 (1.4)	<0.001
NC (cm)^a^	23.4 (1.3)	25.2 (1.5)	<0.001
Girls	(*n *= 81)	(*n *= 56)	
Age (months)	16.0 (3.1)	16.9 (3.2)	0.037
Body mass (kg)	9834.5 (1237.6)	11,772.6 (1524.7)	<0.001
Length (cm)	78.4 (4.2)	78.7 (4.3)	0.443
BMI (kg/m^2^)	15.9 (0.9)	18.9 (1.4)	<0.001
NC (cm)^b^	22.9 (1.3)	24.7 (1.5)	<0.001

^a^BMI ≤P85, *n *= 81; BMI >P85, *n *= 58

^b^BMI ≤P85, *n *= 80

**Table 2:** Relationship between neck circumference (NC) and age, body mass, length and BMI by sex, Joinville, SC, Brazil, 2013-2014.

Characteristic	NC			
	
	Boys		Girls	
	
	*rho*	*p *value	*rho*	*p *value
Age	0.024	0.778	0.124	0.151
Body mass	0.520	<0.001	0.660	<0.001
Length	0.117	0.172	0.358	<0.001
BMI	0.635	<0.001	0.606	<0.001

**Table 3:** Spearman correlation coefficients (*rho*) of the relation between BMI and neck circumference (NC) by age and sex, Joinville, SC, Brazil, 2013-2014

Age (months)	*n*	BMI-NC	
		
		*rho*	*p *value
Boys			
13−15	65	0.604	<0.001
16−19	44	0.663	<0.001
20−24	30	0.719	<0.001
Girls			
13−15	73	0.642	<0.001
16−19	30	0.419	0.021
20−24	33	0.604	<0.001

**Table 4:** Area under the curve (AUC), frequency (*n*), optimal cutoff points, sensitivities and specificities for neck circumference (NC) associated with excess body mass in three age groups of boys and girls, Joinville, SC, Brazil, 2013-2014.

Age (months)	*n*	AUC (95 % CI)	Cutoff	Sensitivity (%)	Specificity (%)
Boys					
13−15	65	0.796 (0.683−0.909)	23.6	85.0	48.9
16−19	44	0.876 (0.771−0.981)	23.9	81.8	63.6
20−24	30	0.902 (0.000−1.000)	24.0	75.0	92.9
Girls					
13−15	73	0.814 (0.709−0.919)	23.4	73.1	61.7
16−19	30	0.697 (0.509−0.885)	23.5	76.9	52.9
20−24	33	0.796 (0.644−0.947)	23.6	76.5	68.7

*CI *confidence interval

## Conclusion

Our results suggest that NC can also be used to screen risk of excess body mass and upper fat distribution in children aged 13-24 months. However, further studies with a larger sample in order to complement our data will be required.

